# Luminescent Organic Barcode Nanowires for Effective Chemical Sensors

**DOI:** 10.3390/polym11040662

**Published:** 2019-04-11

**Authors:** Jinho Choi, Seokho Kim, Jung Woon Park, Seung Hwan Lee, Young Ju Seo, Dong Hyuk Park

**Affiliations:** Department of Chemical Engineering, Inha University, Incheon 22212, Korea; jinho@inha.edu (J.C.); seokho@inha.edu (S.K.); jungwoon@inha.edu (J.W.P.); 22191298@inha.edu (S.H.L.); 22191294@inha.edu (Y.J.S.)

**Keywords:** barcode, Raman scattering, luminescence, chemical sensor, polymer nanowire

## Abstract

Chemical materials are sometimes harmful to the environment as well as humans, plants, and animals. Thus, high-performance sensor systems have become more important in the past few decades. To achieve pH scale sensing in nanosystems, we applied luminescence polymer nanowires with alumina oxide template method with electrochemical polymerization. We made polymer nanowire barcode by alternately stacking poly(3-methylthiophene) (P3MT) and poly(3,4-ethylenedioxythiophene) (PEDOT) in a nanoporous template. After polymerization, a hydrofluoric acid solvent was used to remove the template, and, for changing the pH scale, we used sodium hydroxide. We measured optical properties of each part of barcode using Raman scattering and photoluminescence and confirmed that only P3MT was changed by alkali treatment.

## 1. Introduction

One-dimensional (1D) nanomaterials have specific properties compared to their bulk or thin film counterpart materials [1,2,3,4]. Combining the significantly enhanced electrical and optical properties of individual nanomaterials is a challenge task; thus, hybrid nanomaterials would be suitable for developing optoelectronic devices and sensors. Polymeric π-conjugated compounds have been widely explored owing to their novel electrical and optical properties. These fascinating properties allow for various technological applications, such as organic light-emitting diodes (OLEDs), organic field-effect transistors (OFETs), organic photovoltaic cells (OPVCs), electrochromic devices, field emission displays, and actuators [5,6,7,8,9,10,11]. Facile and reliable methods for the detection of biological/chemical substances are important for scientific research due to their substantial application in sensor development. A π-conjugated polymer is a good candidate for use in developing bio/chemical sensor because of its electrical and optical capability.

We investigated the optical sensing of chemicals, such as acid and alkali, using luminescent organic barcode nanowires (NWs), through distinct emission intensity and color. Unlike conventional bar codes, which are recognized as differences in optical reflection, organic barcode NWs are recognized by the difference in luminescence characteristics in single NW. It was confirmed that only the P3MT segments selectively changed luminescence color from green to red after the alkali treatment within a few tens of seconds. The barcode NWs were fabricated by alternately stacking poly(3-methylthiophene) (P3MT) and poly(3,4-ethylenedioxythiophene) (PEDOT) materials on a nanoporous anodic alumina oxide (Al_2_O_3_) template via electrochemical polymerization [12,13]. PEDOT forms the highest doping level, compared to any other organic semiconductor, and the doping level of PEDOT remains stable. It implies that the P3MT NWs studied here were lightly doped systems rather than heavily doped PEDOT NWs. It should be noted that the P3MT material might be a better system than the PEDOT one, for a study on the chemical sensing effect in the barcode NWs, because of the stable formation of the doping and easy control of optical property by controlling of doping level due to methyl group. As a result, the emission intensity and color of the PEDOT are not changed, and only the P3MT portion was changed through acid or alkali treatment. Distinct light emission patterns were observed due to serial alternation of the P3MT-nanocompartment (NC) and PEDOT-NC in the same barcode-NWs. The color CCD images of the P3MT and PEDOT NCs in the same barcode-NW were bright green and dark green, respectively, due to HF treatment, which exerted different doping effect. We suggest that the discrete luminescence intensities and colors indicative of the P3MT and PEDOT NCs in the single barcode-NWs could introduce various encoding and decoding patterns. The luminescent organic barcode NWs have significant advantages over conventional detection systems: they provide new optical identification nanosystems due to their alternating distinct luminescence and color. Using various light emitting polymers, which have distinguishable optical properties (e.g., fluorescence color and/or intensity), we can encode some of the data in barcode-NWs, because the doping level of each compartment in the barcode NWs and stacked patterns can be controlled during the fabrication. Under a single-wavelength light source, the barcode-NWs were prepared for detection and identification with a microscope and the spectrometer systems guaranteed accuracy, sensitivity, security, and convenience of decoding. In this study, we used effective chemical sensors with an organic polymer-based system, which can facilitate the rapid identification of small amounts of chemical compounds. These features are attractive because they reduce the detection time and simplify the design recognition system while providing high precision in identifying the optical characteristics.

## 2. Materials and Methods 

### 2.1. Preparation of Luminescent Organic Barcode NWs

The luminescent organic barcode NWs were fabricated by alternately stacking P3MT and PEDOT materials via electrochemical polymerization on a nanoporous AAO template. The electrolyte base solution was prepared using an ionic liquid (1-butyl-3-methylimidazolium hexafluorophosphate (BMIMPF_6_)) as a dopant for the electrochemical polymerization of 3-methylthiophene (3MT) and 3,4-ethylenedioxythiophene (EDOT) monomers. Notably, BMIMPF_6_ enhanced the light emission properties of the polymers by increasing environmental stability and fine controlling the doping states. This is considered to be the most suitable material for use as a dopant in chemical sensors of optical characteristic change [14,15,16]. The solvent used throughout the polymerization was acetonitrile (CH_3_CN). EDOT (99% purified) and 3MT (98% purified) monomers, and BMIMPF_6_ (96% purified) were purchased from Sigma-Aldrich Co. (Darmstadt, Germany) and used without further purification. The molar ratio of the monomer to the dopant was 5:1. 3MT and EDOT were alternately dipped into the electrolyte to obtain alternately stacked P3MT and PEDOT NWs on the same nanoporous template. The length and repeat patterns of the P3MT and PEDOT NW code compartments could be changed by varying the applied current density, polymerization time, and number of dipping times. After the polymerization of the organic barcode NWs, hydrofluoric acid (HF) was used to remove the AAO template.

### 2.2. Measurement of Nanoscale Optical Properties and Structural Characteristics

The morphologies of the organic barcode NWs were determined by scanning electron microscope (SEM; JSM-5200, JEOL, Tokyo, Japan) images. A color charge coupled device (CCD) was used to obtain luminescence images of the NWs with an AVT Marlin F-033C system (*λ*_ex_ = 435 nm, Allied Vision Technologies GmbH, Stadtroda, Germany). For accurate comparison of the brightness of the color CCD images under the same experimental conditions, the light exposure was restricted to 1 s. A laser confocal microscope (LCM) built around an inverted optical microscope (Axiovert 200, Zeiss GmbH, Oberkochen, Germany) was used for obtaining the solid-state reflectance images, photoluminescence (PL) images, PL spectra, and Raman scattering spectra of the organic barcode NWs. An unpolarized argon ion laser with a 488 nm line was used for the LCM PL excitation, and a He–Ne gas laser with a 632.8 nm line was used for the Raman scattering excitation. The spot size of the focused laser beam onto the sample was estimated to be about 200 nm. The power of the incident laser and the exposure time for each PL spectrum were fixed at 1 μW and 1 s, respectively, during the LCM PL experiments. More detailed procedures for the LCM PL and Raman experiments have been reported previously [12,13].

## 3. Results

### 3.1. Formation of Organic Barcode NWs of P3MT/PEDOT 

**Figure 1:**
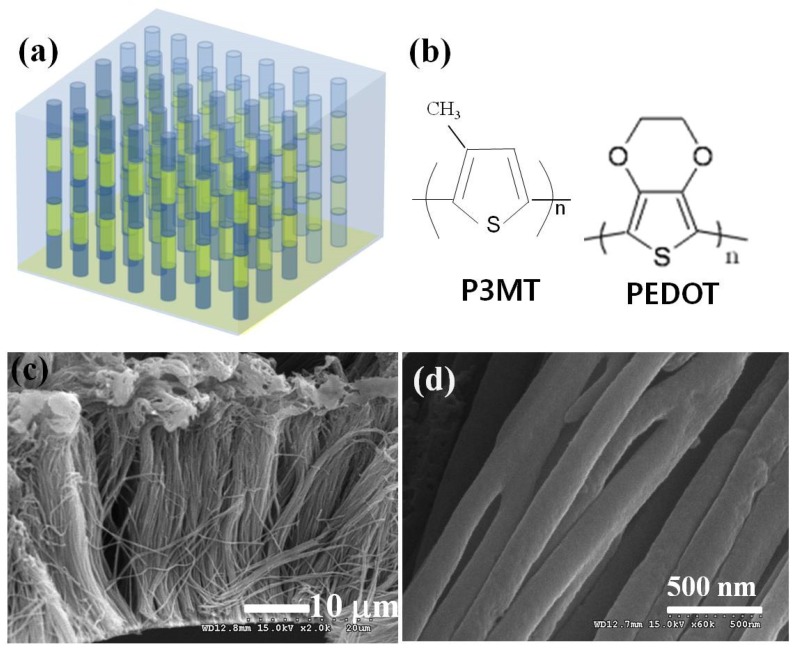
(**a**) Schematic illustration of luminescent organic barcode nanowires. (**b**) Molecular structures of poly(3-methylthiophene) (P3MT) and poly(3,4-ethylenedioxythiophene) (PEDOT). SEM images showing the side (**c**) and top (**d**) view of the luminescent organic barcode NWs.

The organic barcode NWs arrays were fabricated through alternate stacking of P3MT and PEDOT on a conventional nanoporous AAO template by electrochemical polymerization. The formation and surface morphologies of the P3MT–PEDOT barcode-NWs were visualized by SEM. Figure 1a presents the schematic diagrams of the luminescent organic barcode NW array consisting of two different light-emitting polymer NWs on a nanoporous template. Figure 1c shows the homogeneous array of the flexible polymer NWs. Figure 1d shows the uniform diameters (210 ± 20 nm) of the barcode NWs. The SEM observations revealed that the fabricated organic barcode NWs seemed to be a whole identical material without the boundary of different compartments in organic barcode NWs.

**Figure 2:**
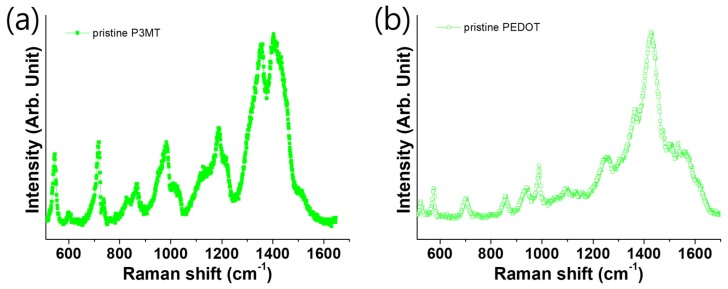
The Raman shift spectra of the (**a**) P3MT and (**b**) PEDOT nanowires excited by a HE-NE laser at 632.8 nm.

However, we found alternating P3MT and PEDOT materials from the LCM Raman spectra analysis of an isolated single NW, as shown in Figure 2. The Raman characteristic peak at 1415 cm^−1^ corresponded to the C=C inter-ring stretching of P3MT, while the peaks at 577 and 990 cm^−1^ corresponded to the oxyethylene ring deformation of PEDOT. These peaks were alternately observed through the scanning using a focused laser and detector along a single NW in the LCM Raman experiments, as shown in Figure 2a,b, respectively.

### 3.2. Luminescence Characteristics of Organic Barcode NWs 

**Figure 3:**
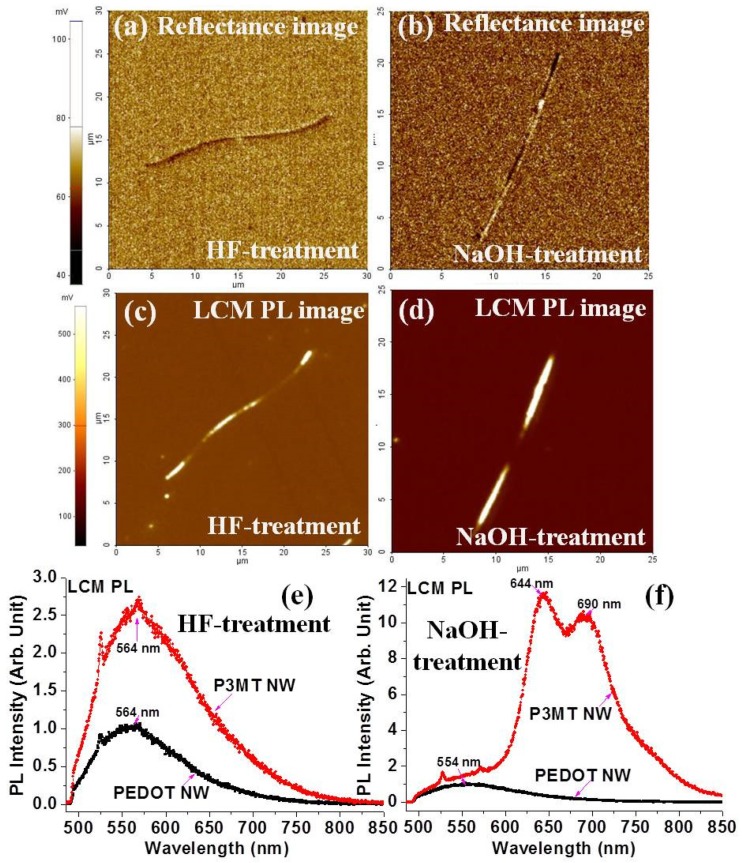
Reflectance images of: (**a**) HF-treated barcode NW; and (**b**) NaOH-treated barcode NW. Two-dimensional LCM PL images of: (**c**) HF-treated barcode NW; and (**d**) NaOH-treated barcode NW. LCM PL spectra of: (**e**) HF-treated barcode NW; and (**f**) NaOH-treated barcode NW.

The luminescence characteristics of an isolated single P3MT–PEDOT barcode-NWs, such as reflectance image and PL properties, were investigated through nanoscale LCM experiments, as shown in Figure 3. There were no significant differences in the reflection images of the NWs treated with HF and NaOH, as shown in Figure 3a,b, respectively. It was confirmed that the organic barcode NW could not be recognized by its reflection characteristics in contrast to a normal barcode. Figure 3c,d shows two-dimensional (2-D) LCM PL images of single unit of the HF-treated and NaOH-treated organic barcode NWs, respectively. Alternating bright and dark luminescence characteristics of P3MT and PEDOT in a single unit of the HF-treated barcode NW were clearly observed in the 2-D LCM PL image, as shown in Figure 3c. The luminescence efficiency of P3MT was different from that of PEDOT. The relatively bright emissions in Figure 3c originated from the luminescent nature of P3MT. The measured voltages corresponding to the LCM PL intensities for the P3MT and PEDOT NWs were 285 ± 30 mV and 73 ± 8 mV, respectively. We could directly observe the different LCM PL intensities of the P3MT and PEDOT NWs in the same barcode NW. These differences were due to the variations in the doping levels and chemical structures. In addition, the luminescence efficiency of the P3MT NWs could be controlled through NaOH treatment (by de-doping), as shown in Figure 3d. The measured voltages corresponding to the LCM PL intensities of the NaOH-treated P3MT and PEDOT NWs in the single barcode NW were 1.9 ± 0.3 V and 80 ± 12 mV, respectively. The measured voltages for the LCM PL intensities of the NaOH-treated P3MT NWs were 5–7 times higher than those of the HF-treated P3MT NWs, while the emission intensity of the PEDOT NWs did not change notably. Figure 3e,f shows the LCM PL spectra of the HF-treated and NaOH-treated P3MT and PEDOT NWs, in the same single barcode NW. LCM PL peaks for the P3MT and PEDOT compartments in the same single NW were detected at ~564 nm (i.e., green light emission) and ~564 nm (i.e., green light emission), respectively. The main LCM PL peak positions were the same, and the PL peak intensities of the P3MT NWs were approximately 2.8 times higher than those of the PEDOT NWs in the same barcode NW. The LCM PL peaks for the NaOH-treated P3MT and PEDOT NWs in the same single NW were detected at ~640 nm (i.e., red light emission) and ~564 nm (i.e., green light emission), respectively. The LCM PL peak position was redshifted and the intensity of the P3MT NWs was ~12 times higher than that of the PEDOT NWs in the same barcode NW. The results indicate that the optical identification sensitivity of the P3MT–PEDOT barcode NWs could be considerably enhanced through NaOH treatment, due to the effects of de-doping on P3MT [17,18].

**Figure 4:**
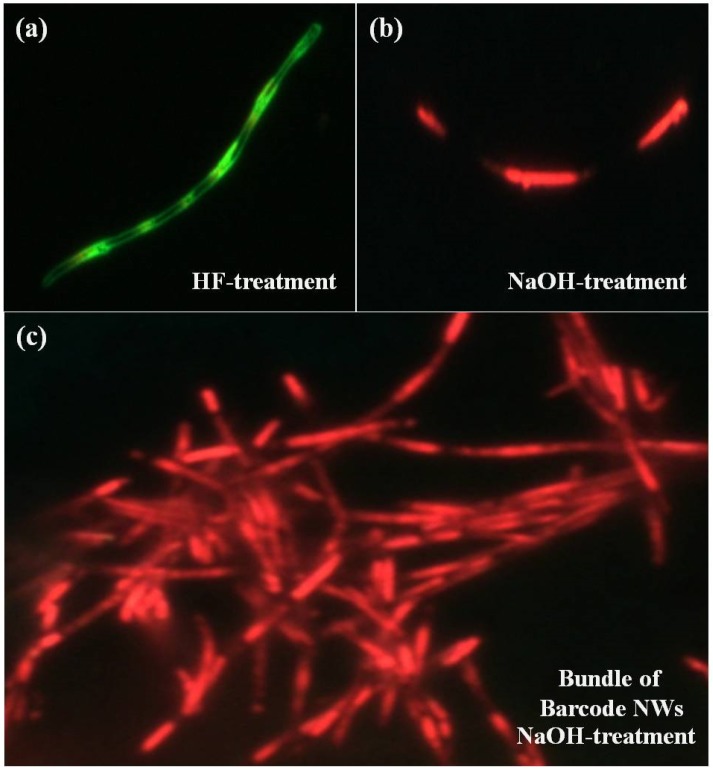
Luminescence CCD images of a single unit of: (**a**) HF-treated barcode NW; (**b**) NaOH-treated barcode NW; and (**c**) bundle of NaOH-treated barcode NW.

Figure 4a,b shows the color CCD images of the HF-treated and NaOH-treated P3MT–PEDOT barcode NWs, respectively. Distinct light emission patterns were observed due to the alternation of P3MT NWs and PEDOT NWs in the same barcode NW. The color CCD images of the P3MT and PEDOT NWs in the same organic barcode-NW were bright green and dark green due to HF treatment, which exerted different doping effects, as shown in Figure 4a. The emission color of P3MT treated with NaOH varied greatly from green to red. Consequently, we could identify the barcode NW where the red and green emissions from the P3MT and PEDOT NWs could be alternately observed in a single barcode NW. These results imply that the signal photo-bleaching effect between the light-emitting polymers was minor due to the high luminescence efficiency of the P3MT NWs. We suggest that discrete luminescence colors due to the P3MT and PEDOT NWs in the single barcode NWs can be introduced for effective chemical sensors. The color CCD image of the bundle of NaOH-treated P3MT–PEDOT barcode NWs is shown in Figure 4c. We consistently observed distinct luminescence characteristics, i.e., alternating red and green emissions, for the whole organic barcode NWs. These results indicate that the NP3MT–PEDOT barcode NWs are promising optical barcode NWs in terms of their highly distinct emission, with control of the luminescence color and intensity for identification and detection.

### 3.3. Raman Scattering of P3MT and PEDOT with Difference pH Scale

**Figure 5:**
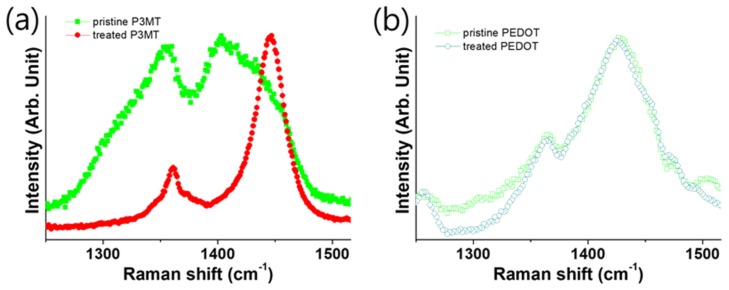
Magnified Raman shift spectra between 1200 and 1550 cm^−1^. (**a**,**b**) P3MT and PEDOT nanowires prepared in different pH environments (square-dotted line: pristine, circle-dotted line: alkali treated).

The LCM Raman characteristic peaks of the P3MT NW in the range of 1050–1600 cm^−1^ showed significant changes after the NaOH treatment. The intensities of the LCM Raman characteristic peaks at 1214, 1352, and 1390 cm^−1^ decreased considerably, as shown in Figure 5a. The Q1 and Q2 peaks also decreased dramatically after the NaOH treatment. We suggest that the NaOH-treated P3MT NWs in the barcode NWs might have induced the conformational modification of the P3MT chains and the de-doping states of the P3MT NWs [18].

The molecular structure of doped PEDOT changes from a benzoid form to a quinoid form, as indicated by the Raman peaks. The Raman peak at 1428 cm^−1^ was indicative of the benzoid PEDOT, while that indicative of quinoid PEDOT showed a redshift. In the polymerization, we used HF to eliminate the AAO template and afford the polymer NWs. Subsequently, we modified the pH of PEDOT from acidic to alkaline using NaOH. Figure 5b shows the Raman peaks of the pristine PEDOT untreated with alkali; we observed a peak indicative of doping at 1428 cm^−1^. Additionally, we found the Raman peaks indicative of the alkali-treated PEDOT NWs at the same position (1428 cm^−1^) in Figure 5b, because the doping state of quinoid PEDOT was unaffected by alkali treatment [19,20,21]. 

**Figure 6:**
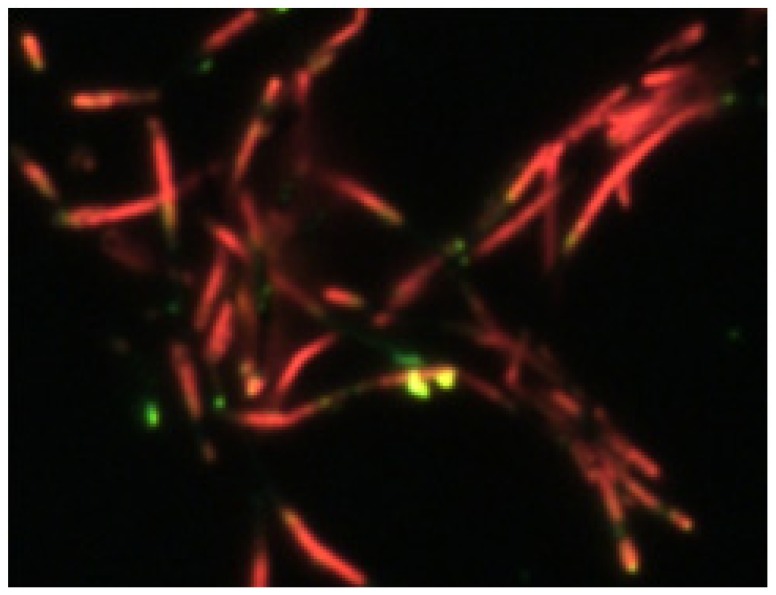
Luminescence CCD images of bundle of ammonia vapor-treated barcode NW.

We carried out further experiments to confirm the possible practical application of the organic barcode NWs to chemical sensor on the ammonia vapor treatment. The color CCD image of the bundle of ammonia vapor-treated P3MT–PEDOT barcode NWs is shown in Figure 6. The change of the luminescence characteristics was observed through the treatment with ammonia in the highly doped barcode NWs. It was confirmed that only the P3MT segments selectively changed luminescence color from green to red after the treatment with ammonia vapor treatment within a few tens of seconds. Therefore, we conclude that the unique chemically responsive luminescent organic NWs may be applied as effective chemical sensors through fast and sensitive response.

## 4. Conclusions

Luminescent color barcode NWs combined with two different luminescent polymers, P3MT and PEDOT, were fabricated by electrochemical polymerization. Distinct light emission patterns were observed due to alternation of the P3MT NWs and PEDOT NWs in the same barcode NW. Furthermore, the optical detection ability of the barcode NWs was improved through a selective de-doping process. We suggest that the discrete luminescence intensities and colors due to the P3MT and PEDOT NWs in a single barcode NW can introduce various encoding and decoding patterns. The luminescent organic barcode NWs provide significant advantages over conventional detection systems and thus offer new optical identification nanosystems due to the distinct alternating luminescence and colors. With various combinations of distinguishable luminescence intensities and colors in each compartment using different light-emitting polymers, encoding and decoding in the barcode NWs are possible for effective chemical sensing.
